# Curcumin and Endometriosis

**DOI:** 10.3390/ijms21072440

**Published:** 2020-03-31

**Authors:** Alexandre Vallée, Yves Lecarpentier

**Affiliations:** 1 Diagnosis and Therapeutic Center, Hypertension and Cardiovascular Prevention Unit, Hôtel-Dieu Hospital, AP-HP, Paris-Descartes University, 75004 Paris, France; 2 Centre de Recherche Clinique, Grand Hôpital de l’Est Francilien (GHEF), 77100 Meaux, France; yves.c.lecarpentier@gmail.com

**Keywords:** endometriosis, curcumin, inflammation, oxidative stress

## Abstract

Endometriosis is one of the main common gynecological disorders, which is characterized by the presence of glands and stroma outside the uterine cavity. Some findings have highlighted the main role of inflammation in endometriosis by acting on proliferation, apoptosis and angiogenesis. Oxidative stress, an imbalance between reactive oxygen species and antioxidants, could have a key role in the initiation and progression of endometriosis by resulting in inflammatory responses in the peritoneal cavity. Nevertheless, the mechanisms underlying this disease are still unclear and therapies are not currently efficient. Curcumin is a major anti-inflammatory agent. Several findings have highlighted the anti-oxidant, anti-inflammatory and anti-angiogenic properties of curcumin. The purpose of this review is to summarize the potential action of curcumin in endometriosis by acting on inflammation, oxidative stress, invasion and adhesion, apoptosis and angiogenesis.

## 1. Introduction

Endometriosis is one of the main common gynecological disorders, which is characterized by the presence of glands and stroma outside the uterine cavity [1]. Between 6% and 10% of women in a reproductive age are affected by this disease. The primary symptoms of endometriosis are pelvic pain and infertility. Other symptoms are dysmenorrhea, irregular uterine bleeding, dyspareunia and dysuria [2,3]. Endometriotic lesions are often detected in the ovaries, fallopian tubes, the ligaments of the uterus, the cervical–vaginal area, the abdominal wall and umbilicus, the urinary tract and the rectum [4,5]. Environmental, endocrine, genetic and immunological factors have been observed in the initiation of endometriosis and, thus, its development [6,7]. Some findings have highlighted the main role of inflammation in endometriosis by acting on proliferation, apoptosis and angiogenesis [1]. Furthermore, oxidative stress (OS), an imbalance between reactive oxygen species (ROS) and antioxidants, could have a key role in the initiation and progression of endometriosis by resulting in inflammatory responses in the peritoneal cavity [8,9].

Hormone therapy, medication and surgery are used to eradicate the symptoms in endometriotic patients. Pain-relieving, non-steroidal anti-inflammatory drugs, aromatase inhibitors, progestins, combined estrogen–progestin therapy and selective progesterone receptor modulators are the main common recommended therapies [5,10]. Nevertheless, the mechanisms underlying this disease are still unclear and therapies are not currently efficient. The introduction of new agents can be effective in improving the condition of patients; for example, plants are promising sources of bioactive natural components [11]. These natural compounds could be interesting strategies in therapy. Curcumin (1,7-bis(4-hydroxy-3-methoxyphenyl)-1,6-heptadiene-3,5-dione) is a natural product that presents polyphenolic phytochemical properties from the rhizome of *Curcuma longa* L. [12]. Curcumin has been discovered in 1815 by Vogel and Pelletier [13]. Its yellow-colored hydrophobic component is traditionally used in Asian countries for its several properties against pathophysiological states, including being anti-cancer [14]. Curcumin is a major anti-inflammatory agent. Studies have shown inconsistent results regarding the effects of curcumin in different diseases [15,16,17,18,19]; but up to now, the anti-oxidant, anti-inflammatory and anti-angiogenic properties of curcumin have been reported in several animal and human studies [20,21,22]. Curcumin decreases the inflammation in diseases, including cancers. Some studies have shown the role of curcumin in the prevention and the treatment of various cancers, including gastrointestinal, respiratory, lymphatic, skin and reproductive systems [23]. Curcumin use may have a major role in the control of inflammation, cell proliferation and angiogenesis [24]. The purpose of this review is to summarize the potential action of curcumin in endometriosis by acting on inflammation, oxidative stress, invasion and adhesion, apoptosis and angiogenesis.

## 2. Endometriosis

Endometriosis is a disease initiated by the growth of endometriotic glands and stroma outside of the uterus. Chronic pelvic pain and infertility can affect 10% of women [25]. Some symptoms, including dyspareunia, dysuria, dysmenorrhea and pain, characterize endometriosis. Nevertheless, the diagnosis of this disease remains uncommon [26]. Presence of endometrial tissue in ectopic lesions are associated with lymphatic/vascular metastases, celomic metaplasia and retrograde menstruation [27]. The underlying pathophysiology of endometriosis remains unclear. Endometriotic deposits have been found within the pelvis, the peritoneal surfaces of pelvic organs and within the pelvic peritoneum [28]. Organs affected by this disease are the uterus, ovaries, appendix, fallopian tubes, rectum, bladder and ureters. The deposits are named endometriomas due to old blood products. The pelvic ligaments, posterior cul-de-sac, rectovaginal septum and vesicouterine space can be also involved in endometriosis. Other deposits have been found outside the pelvis [29]. The diagnostic gold-standard of endometriosis remains laparoscopy, which is preferable to histologic confirmation [28]. Recent findings have highlighted the interest of ultrasound and MRI in the diagnosis of endometriosis [30].

## 3. Pathophysiology of Endometriosis

### 3.1. Inflammation

Inflammation has a main role in the progression of endometriosis [31]. The cascade of the different markers of inflammation leads in the upregulation of metalloproteinases, prostaglandins, cytokines and chemokines [4]. These mediators have been shown to be upregulated in peritoneal serum of endometriosis patients and in the endometrium [32,33,34]. In contrast, healthy cells of endometrium did not present this phenomenon [35]. Interlieukin-10 (IL-10, IL-6, IL-8, COX2 (cyclooxygenase-2), VEGF (vascular endothelial growth factor) and tumor necrosis factor α (TNF-α) have been observed to be increased in the peritoneal fluid of endometriosis [34,36]. The stroma of the endometrium is associated with adhesion of extracellular matrix proteins while IL-8 and matrix metalloproteinase (MMP) have been increased [37].

In parallel, ROS production in endometriosis leads to the over-activation of the NF-κB pathway by stimulating angiogenesis, cell growth, inflammation and molecule adhesion [38]. Moreover, the initiation of endometriosis is associated with the upregulation of the NF-κB pathway, suggesting its role in cell growth, proliferation and apoptosis [39].

### 3.2. Oxidative Stress

Oxidative stress (OS) occurs because of an imbalance between ROS production and antioxidants. ROS are molecules having an unpaired electron and that are stabilized by themselves to extract electrons from some molecules in the body, such as lipids, nucleic acids and proteins.

Antioxidants are defense pathways elaborated by human to inhibit ROS production. ROS production presents a physiological interest in the body with respect to reproduction. Macrophages and apoptotic endometrial tissue transplanting into the peritoneal cavity, possibly by retrograde menstruation, are thought to be inducers of OS with endometriosis in women. Endometriosis and cancer have some common characteristics, including a tendency to invade tissues, an uncontrolled growth, angiogenesis processes and an ability to avoid apoptosis [40]. The long-term survival and proliferation of both endometriosis lesions and cancer cells are critically reliant upon adequate blood supply by angiogenesis and apoptosis protection. A well-established correlation between the ROS production, cell proliferation and metastatic character of tumor cells has been shown in many studies [41,42]. In both endometriosis and tumor cells, increased ROS production is associated with an augmented proliferation rate. Likewise, OS-mediated damages in the pathogenesis of endometriosis and tumor cells are similar [41]. ROS production serves as inductor of cell proliferation [43]. Increased ROS production is associated with cell proliferation through the activation of the mitogen-activated protein kinases (MAPK) pathway. The well-described association between ROS production and proliferation of tumor cells points towards ROS as a major role player in the regulation of cell proliferation in endometriosis.

ROS production enhances NF-κB in peritoneal macrophages leading to cell growth, angiogenesis and inflammation in endometriosis cells [44].

### 3.3. Angiogenesis

Angiogenesis is characterized by the growth of new capillaries through proliferation and migration of preexisting differentiated endothelial cells. Angiogenesis acts in both embryonic initiation and postnatal life [45,46]. Numerous pathways are involved in the different angiogenesis processes [47]. The dysfunction of growth factors plays a major role in angiogenesis [48]. VEGF may be due to physiological activators, including inflammation and hypoxia [49,50]. The hypoxia-inducible factor 1 α (HIF-1α)/VEGF pathway enhances endothelial cell proliferation and migration [51].

Angiogenesis is defined by many steps: blood vessel breakdown, basement membrane degradation, surrounding extracellular matrix (ECM), endothelial cells migration and new blood vessels formation [52].

From existing vessels, new blood vessels are formed by the dissolution of aspects of native vessels. Angiopoietin-1 and 2 (ANG-1 and ANG-2) are major endothelial growth factors acting through TIE-2 receptor tyrosine kinase (RTK) expressed in endothelial cells. Under physiological conditions, ANG-1 links TIE-2 to induce an association between pericytes and endothelial cells, to stabilize the vasculature [53,54]. ANG-1 operates as a stimulator ligand for TIE-2 while ANG-2 downregulates TIE-2 phosphorylation, even in the presence of ANG-1 [55,56]. TIE-2 is a key factor of the physiological vascular development [57]. TIE-2 is a main factor of the mature vasculature homeostasis. ANG-2 is an antagonist of TIE-2 phosphorylation, which leads to destabilizing the structure of blood vessels [56,58]. In the presence of ANG-2, VEGF promotes migration and proliferation of endothelial cells and stimulates the growth of new blood vessels [59].

The angiogenesis process is composed of the dysregulation of the vessel basement membrane and the surrounding ECM [60]. The MMP enzymes family degrade components of ECM by collagenases, gelatinases, stromelysins and membrane-associated MMPs. Gelatinase-A (MMP-2) and gelatinase (MMP-9) are present in blood vessels. MMP-2 and MMP-9 have synergistic effects on the basement membrane degradation [61].

Angiogenesis has been reported in endometriosis whereas its underlying processes are still unclear in this disease [62].

### 3.4. Adhesion and Invasion

In endometriosis, molecule adhesion enhances the attachment of endometrial-like tissues to ectopic sites [63]. Numerous findings suggest that ectopic endometriotic cells have the ability to invade their surrounding environment and can metastasize in lymph nodes and in the abdominal cavity [64].

### 3.5. Apoptosis

Homeostasis maintenance of tissue is mainly regulated by cell death. A balance between cell proliferation and cell apoptosis maintains homeostasis in cells against diseases. Some studies have shown that apoptosis increases during the menstrual cycle to retain cell homeostasis to remove aged cells from the functional layer of the endometrium [65,66]. The reduction in cell death in endometriosis could be causal for the initiation of this disease [67,68]. The rate of apoptosis is decreased in endometrial cells of women endometriosis [69]. Moreover, the activated NF-κB pathway in endometriosis is associated with cell proliferation and apoptosis [70,71].

### 3.6. Circadian Dysregulation

Few studies have focused on the important role of the circadian clock in endometriosis. Period 2, a clock gene, can control the expansion of human endometrial stromal cells [72]. Period 2 is a main marker of the transition from oscillatory receptivity to the non-oscillatory decidual endometrium [73]. Its inhibition is marked by a transitional phase with increased ROS production, an altered redox pathway and increased expression of decidual marker genes [74,75]. The modification of Period 2 expression is a response to hormonal dysregulation [76,77]. Period 2 binds estrogen receptor-alpha and inhibits estrogen-dependent proliferation in breast cancer cells [78,79]. Period 2 can promote or inhibit the cell cycle progression by a dependent hormonal regulation [72].

## 4. Curcumin

The use of dietary supplements and nutraceuticals has gained popularity over the few decades due to the increased role of, and thus interest in, natural products [80]. Curcumin, defined as bis-α, β-unsaturated β-diketone, is a natural component well documented since 1815. Curcumin is the active compound of turmeric or *Curcuma longa* L. and presents a surprising wide range of beneficial properties, such as anti-cancer features [81]. Curcumin presents some therapeutically potential roles as anti-inflammatory, anti-cancer and anti-aging [82]. In 1815, curcumin has been isolated by Vogel and Pelletier from the rhizomes of *C. longa* [83]. For the first time, in 1842, Vogel Jr purified curcumin. In 1910, Melabedzka et al. presented the structure of curcumin as diferuloylmethane, or 1,6-heptadiene-3,5-dione-1,7-bis (4-hydroxy-3-methoxyphenyl)-(1*E*,6*E*) [83]. In 1913, Lampe and Melobedzka have shown a method to synthesize curcumin [84]. In 1953, Srinivasan showed, by chromatography separation and quantification, the different components of curcumin [85].

The health benefits of curcumin are limited by its poor oral bioavailability that could be attributed to poor absorption, high metabolism rate and rapid systemic increase in the body. Curcumin is converted into its water-soluble metabolites and then excreted in the urine. This metabolism consists of two stages. First, a reduction in the metabolism dependent on NADPH, including the reduction of the double bonds of the heptadiene-3, 5-dione structure catalyzed by curcumin reductase dependent on NADPH. Secondly, a conjugation process was observed with monoglucuronide via a glucuronidase. These mechanisms are responsible for the low solubility and rapid metabolism of curcumin.

Although some studies have found that curcumin pharmacokinetics have shown low bioavailability [86], strong pharmacological and clinical applications have been reported [87]. Nevertheless, some of the possible ways to overcome this poor bioavailability can be counteracted by these aspects. Strategies can improve this bioavailability, such as phospholipid complexes, liposomes and nanoparticles. Some polymers have been used to prepare nanoformulations for curcumin administration to improve its biological activity [88]. Biocompatible and biodegradable polymers are used in drug delivery systems because of their low toxicity risk [89]. Advances in liposome formulations have resulted in improved treatment of drug-resistant tumors and reduced toxicity [90]. Liposomes are made by phospholipid bilayer shells and watery nuclei, resulting in the encapsulation of curcumin by both hydrophobic and hydrophilic components. Other curcumin delivery systems are used, such as nanogels [91], peptide and protein formulations [92] and cyclodextrin complexes [93].

## 5. Actions of Curcumin in Endometriosis

### 5.1. Curcumin and Inflammation

In endometriosis cells, the NF-κB pathway, one of the main markers of inflammation, can regulate the proliferation, apoptosis and inflammation processes observed [94]. In normal endometrium, the NF-κB pathway is downregulated [95] whereas its expression is increased under the various endometriosis stages [96]. An activated NF-κB pathway leads to the formation of the complex NF-κB–IkappaB to activate its nuclear translocation [97]. This complex activates IL-6 and IL-8 in endometriosis [94] while its inhibition could be associated with the reduction of endometriosis development [98]. In endometriosis, NF-κB pathway activation is associated with better cell survival, growth and inflammatory processes [99].

Many studies have shown the value of curcumin in inflammation [100,101]. Curcumin administration can reduce the activity of the NF-κB pathway [102]. In parallel, curcumin can downregulate the expression of TNF-α, COX-2, IL-6 and TGF [103]. Curcumin has been shown to decrease inflammation by inhibiting the expression of inflammatory factors, such as the NF-κB pathway, TNF-α, IL-1, IL-6 and IL-8, in mice macrophages [104,105].

Moreover, NF-κB activation is inhibited by curcumin through the blockage of I-κB phosphorylation [106] and the inactivation of the I-κB kinase complex [107]. AP-1 controls the expression of pro-inflammatory factors and antioxidant genes. The inhibition of AP-1 could be due to an action of curcumin with AP-1 and due to the inhibition of its components c-Jun and c-fos [22].

Activation of COX-2 is associated with cell proliferation and the suppression of apoptosis [108]. Some studies have shown that curcumin can inhibit COX-2 expression in animal models and cell cultures [109,110,111]. Curcumin can target the TNF-α expression to improve the growth differentiation factor-9 (GDF-9) expression in peritoneal fluid of women with endometriosis [112]. A recent study has shown that curcumin can be an interesting treatment of endometriosis by abrogating the aberrant activation of chemokines, cytokines and the NF-κB pathway [113] (Table 1).

### 5.2. Curcumin and Oxidative Stress

OS is considered as one of the main determinants of the endometriosis process [124] (Table 1). Some studies have shown that prevention of endometriosis could be done by administration of antioxidants [125]. OS damages observed in endometriosis cells are localized in the proliferation process [126]. Superoxide dismutase activity is higher in endometriosis cells compared to healthy cells [127]. In endometriosis cells, OS can enhance the proliferation process [43] through the activation of MAPK/extracellular signal-regulated kinase (ERK), which acts on the proliferation and survival cell process. The ERK pathway is activated by ROS to enhance the proliferative response [128].

In endometriosis, there are few reports on the role of curcumin in OS. Nevertheless, administration of Letrozole–curcumin has been associated with a reduction of ROS serum and lipid peroxidation in mice [114]. Moreover, therapy by curcumin in endometriosis mice can prevent against lipid peroxidation and protein oxidation [115]. Curcumin treatment can diminish OS by regulating the Nrf2-Keap1 pathway [102].

Some evidence suggests that the curcumin antioxidant activity is comparable to vitamins C and E [129]. Curcumin can scavenge free radicals, such as ROS and nitrogen dioxide radicals [129,130,131]. Curcumin can inhibit lipid peroxidation in animal models [132] and the expression of nitric oxide (NO) synthase in mice macrophages [116], and can increase glutathione and superoxide dismutase in lymphocytes [117].

### 5.3. Curcumin and Angiogenesis

Experimental studies have shown an interest in using anti-VEGF factors to inhibit the growth of endometriosis without impacting ovarian function [133] (Table 1). VEGF activation involves the stimulation of the PI3K/Akt pathway [134]. This activated pathway contributes to the initiation of angiogenesis and inhibition of apoptosis [135]. By activating HIF-1α and cyclin D1, the PI3K/Akt pathway makes angiogenesis without hypoxia possible [135,136]. The downregulation of NME1 in the endometrium leads to activation of the PI3K/Akt pathway and to an increase in the expression of VEGF and IL-8, inducing production of new vascular cells in ectopic endometrial lesions [137]. The elevation of the PI3K/Akt pathway in endometriosis could be associated with NOS expression and OS [138]. By maintaining the fibrotic environment of endometriosis, the PI3K/Akt pathway activates the ERK pathway [139]. Moreover, the PI3K/Akt pathway activates the NF-κB pathway, a main activator of VEGF, to stimulate cell proliferation and angiogenesis in endometriosis [140].

Few studies have focused on the role of curcumin on angiogenesis in endometriosis. Curcumin reduces VEGF expression in ectopic endometrium but not in eutopic endometrium [118]. However, curcumin can reduce the expression of VEGF in ovarian cancers [119]. Curcumin administration is associated with the decrease of HIF-1α in tumors cells [135,141].

### 5.4. Curcumin and Invasion and Adhesion

The endometriotic peritoneum of women presents an overproduction of MMP, especially MMP-1, 2, 3, 9 and 11, and cellular adhesion molecules, including ICAM-1, integrins and cadherins. Molecules adhesion plays a main role in tissue attachment and, then, in the invasion of ectopic lesions. MMPs have a major role in implant progression and angiogenesis [120,142] (Table 1). MMPs are implicated in numerous reproductive processes, including menstruation, ovulation and embryo implantation [143,144]. MMP endometrial expression is low in the proliferative step, it declines in the early secretory step, but increases in the late secretory step. Progesterone is one of the major inhibitors of MMP expression while MMPs are controlled by different hormones, cytokines and growth factors. Progesterone can regulate the MMP expression by the plasminogen activator pathway that enhances the levels of plasminogen activator inhibitor (PAI)-1 and decreases the plasmin-mediated expression of latent MMP [145,146,147]. The production of retinoic acid and transforming growth factor-β (TGF-β) enhances the expression of tissue inhibitors of metalloproteinases (TIMPs). MMP activity is the initial mediator of maintenance and survival of lesions [148].

Curcumin action on cell invasion has been shown in several studies [100]. Curcumin administration can decrease MMP-2, MMP-3 and MMP-9 in serum mice presenting endometriosis [120,121]. Curcumin can downregulate the expression of mRNA and proteins expressing intracellular adhesion molecule 1 (ICAM-1) and vascular cell adhesion molecule 1 (VCAM-1) in a dose-dependent manner [94]. In endometriosis lesions, curcumin inhibits MMP-9 expression and can attenuate TIMP-1 expression [115]. In parallel, MMPs expressions are inhibited by curcumin in several cell types [22,149]. Curcumin decreases the activity of both MMP-2 and MMP-9 in human fibrosarcoma cells [122]. Curcumin can diminish MMP-9 expression in human intestinal epithelial cells [150], orthotopically implanted pancreatic tumors [109], ovarian tumors in nude mice [119] and can inhibit the production of MMP-3 in primary human colonic myofibroblasts [151]. Some studies have suggested that curcumin can decrease the expression of adhesion molecules, such as endothelial-leukocyte adhesion molecule 1 (ELAM-1), ICAM-1, and VCAM-l in endothelial cells and in orthotopically implanted pancreatic tumors in mice to reduce cell adhesion and invasion [109,152].

### 5.5. Curcumin and Apoptosis

The PI3K/Akt pathway is activated in endometriosis cells [153] and leads to apoptosis [154] (Table 1). A vicious circle operates between NF-κB and PI3K/Akt to stimulate apoptosis [155]. The NF-κB pathway inhibits the antiapoptotic role of the PI3K/Akt pathway [156]. X-linked inhibitor of apoptosis protein (XIAP) operates as caspase-3 and caspase-9 inhibitors and regulates the Bax-cytochrome c pathway leading to an apoptosis mechanism through the inhibition of caspase-9 [157]. The PI3K/Akt signaling pathway involves the stimulation of both XIAP and B cell lymphoma extra-large (Bcl-xL) expressions. Moreover, Bcl-xL is activated by the PI3K/Akt pathway [158,159]. In ectopic endometriosis tissue, B-cell lymphoma 2 (BCL-2) presents some modulatory roles in apoptosis and cell proliferation [160]. Bcl-2 can induce antiapoptotic features [161]. In normal conditions, activation of ERK1/2 stimulates the cell proliferation and the promotion of angiogenesis [162,163]. ERK1/2 stimulation can downregulate the expression of Bcl-2, leading to dysregulate mitochondrial-dependent cell death [164]. The PI3K/Akt and MAPK pathways lead to anti-apoptosis action in endometriosis [153,165].

A crosstalk operates between the ERK pathway and PI3K/Akt pathway [166,167]. The inhibition of the PI3K/Akt pathway leads to the activation of the ERK pathway [168] and the reciprocity is verifiable [169]. Some studies have shown the interest of co-targeting these two pathways in endometriosis [43].

Recent studies have shown that curcumin decreases the number of endometriosis stromal cells and the process of cell growth in a dose-dependent manner [170]. In BALB-mice, curcumin administration is associated with the reduction of endometriosis progression and the activation of apoptosis [121]. Curcumin administration leads to an increase in the ratio of pro-apoptotic factor Baw/anti-apoptotic factor Bcl-2, the induction of cytochrome c and caspase 9 and tumor suppressor protein p53 [121]. Moreover, curcumin decreases the weight and volume of endometriotic tissues in a dose-dependent manner in experimental rat models [118]. Nevertheless, it induces no effect in human endometriotic stromal cells [94]. In a dose-dependent manner, curcumin decreases the expression of the anti-apoptotic factor Bcl-2 [123] and diminishes the expression of mRNA and protein expression of Bcl-2 in endometrial cells of mice [171]. In parallel, curcumin inhibits cell proliferation and activates apoptosis in endometrial tumor cells [172]. Moreover, curcumin modulates the expression of VEGF [173], releases cytochrome c, stimulates caspase-8 expression and reduces Bcl-2 and cyclin-D1 expressions [104,174,175,176,177].

### 5.6. Curcumin and Melatonin

Some studies have shown that endometriosis is associated with low levels of melatonin [178]. Moreover, endometrial biopsies have presented variations in the expression of melatonin receptors (MR1A and B) depending on the ectopic tissue site [179]. The use of melatonin in endometriosis reverses the lipoperoxidation and decreases antioxidants activities observed after pinealectomy of rat models [180]. Melatonin leads to the reduction in endometriotic foci and histopathologic scores with increased levels of SOD [181]. SCID animals which received melatonin present a reduction in endometriosis lesions in oophorectomized rats [182]. This melatonin-induced decrease in endometriotic lesions is correlated with the downregulation of COX-2 levels [183]. Activities of SOD and TIMP-2 are higher after melatonin treatment while VEGF and MMP-9 levels are reduced [184]. Melatonin can lead in the regression of apoptosis through the caspase-3 pathway [185]. Moreover, treatment by melatonin results in the reduction of plasma levels of luteinizing hormone and estradiol, the promotion of differential regulation of estrogen, progesterone and androgen receptors [186]. Melatonin receptors have been observed in rat uterine endometrium, which suggests that melatonin can have a major role in the physiology of this disease [187]. The proliferation of endometrial cells can be downregulated by MT1 receptor-targeting by melatonin [188].

Few studies have focused on the potential interaction of curcumin and melatonin in endometriosis. However, recent findings have shown that co-treatment of melatonin and curcumin can decrease COX-2 expression and can repress the NF-kappaB pathway [189]. Moreover, this combination can inhibit MMP-2, MMP-9 and TIMP-2 expression [189]. Curcumin can activate sirtuin 1 (SIRT1) [134]. SIRT1 regulates the circadian rhythms. SIRT1 indirectly controls the circadian clock by downregulating the NF-κB pathway [190], inhibiting the nuclear localization of Per2 [191] and the binding to Clock/Bmal1 [192].

## 6. Conclusions

Curcumin can downregulate inflammation and OS in endometriosis. Moreover, curcumin can direct act on invasion, adhesion, apoptosis and angiogenesis in endometrial lesions. The use of curcumin could be interesting in dietary prevention and disease management for women. Nevertheless, the limited number of studies focusing on the different interactions of curcumin in endometriosis restricts its clear and immediate use in a therapeutic strategy. Future clinical trials are needed to better investigate and highlight the role of curcumin in endometriosis.

## Figures and Tables

**Table 1:** Effects of curcumin on inflammation, oxidative stress, angiogenesis and invasion.

Action	Model	Effect	Reference
Inflammation	Nx rats	Reduction of NF-kappaB	[102]
	Rat liver	TNF-α, COX-2, IL-6 and TGF-beta	[103]
	Mice macrophages	NF-κB pathway, TNF-α, IL-1, IL-6, and IL-8	[104,105]
	Human eutopic endometrial stromal cells	chemokines, cytokines and NF-κB pathway	[113]
	Human myeloid ML-1a cells	Blockage of I-κB phosphorylation	[106]
	Intestinal epithelial cells	the inactivation of I-κB kinase complex	[107]
Oxidative stress	Mice	Reduction of ROS serum and lipid peroxidation	[114]
	Mice	Lipid peroxidation and protein oxidation	[115]
	Nx rats	Regulation of NrF2-Keap1	[102]
	Mice macrophages	Diminution of NO synthase expression	[116]
	lymphocytes	Increase of glutathione and superoxide dismutase	[117]
Angiogenesis	Ectopic endometrium	Reduction of VEGF	[118]
	Ovarian cancers	Reduction of VEGF	[119]
Invasion and Adhesion	Serum mice with endometriosis	Reduction of MMP-2, MMP-3 and MMP-9 expression	[120,121]
	Human endometriotic stromal cells	Reduction of ICAM-1 and VCAM-1 expression	[94]
	Endometriotic lesions	Reduction of TIMP-1 expression	[115]
	Fibrosarcoma cells	Reduction of MMP-2 and MMP-9	[122]
Apoptosis	BALB-mice	Increase ratio Baw/Bcl-2, increase cytochrome c and caspase 9	[121]
	Rat experimental models	Decrease of weight and volume of endometriotic tissues	[118]
	Endometriotic cells of mice	Diminution of Bcl-2 expression, diminution of mRNA and protein expression of Bcl-2	[123]

## Abbreviations

IL	interleukin
MMP	matrix metalloproteinase
OS	oxidative stress
RORs	retinoid-related orphan receptors
NF-κB	nuclear factor κB
TNF	tumor necrosis factor
